# Changes in Anterior, Posterior, and Total Corneal Astigmatism after Descemet Membrane Endothelial Keratoplasty

**DOI:** 10.1155/2017/4068963

**Published:** 2017-05-02

**Authors:** Maged Alnawaiseh, Lars Zumhagen, André Rosentreter, Nicole Eter

**Affiliations:** Department of Ophthalmology, University of Muenster Medical Center, Muenster, Germany

## Abstract

*Purpose*. To evaluate changes in anterior, posterior, and total corneal astigmatism in patients after Descemet membrane endothelial keratoplasty (DMEK). *Methods*. We retrospectively included 29 eyes of 23 patients (age 67.6 ± 9.8 years, 13 female, 10 male) after DMEK surgery. The magnitude and axis orientation of anterior, posterior, and total corneal astigmatism before and after DMEK were determined using a rotating Scheimpflug system (Pentacam HR, Oculus). *Results*. The magnitude of anterior, posterior, and total corneal astigmatism in the central cornea did not change significantly after surgery. Before surgery, we found a significant correlation between the magnitudes of anterior and posterior corneal astigmatism (Spearman's correlation coefficient (*r*_S_) = 0.526, *P* = 0.003), while after surgery this correlation was no longer significant (*r*_S_ = 0.038, *P* = 0.843). There was a significant correlation between the vector difference between preoperative and postoperative posterior astigmatism and the change in corneal pachymetry (*r*_P_ = 0.47, *P* = 0.010). *Conclusions*. Posterior corneal astigmatism (especially the orientation) and therefore the relationship between anterior and total corneal astigmatism may change after DMEK. This should be considered to improve the accuracy of toric IOL power calculations following phakic DMEK or in combined procedures.

## 1. Introduction

With advantages such as a lower risk of rejection, faster visual rehabilitation, improved visual outcome, and refractive stability after DMEK [1–5], DMEK surgery is becoming an increasingly popular option for the treatment of Fuchs' endothelial dystrophy (FED) [6, 7]. Cataract surgery can be performed together with DMEK in the same setting [3], or in phakic eyes after DMEK surgery [8].

Cataract surgery and implantation of toric intraocular lenses (IOLs) are often performed to correct corneal astigmatism, resulting in satisfactory spectacle-free visual outcomes. To achieve perfect correction of corneal astigmatism, correct and precise measurements of corneal astigmatism must be obtained to determine the magnitude and meridian [9].

Wacker et al. showed relevant changes in corneal astigmatism based on asymmetric corneal swelling in the course of FED and demonstrated differences compared with the normal population at different stages of the disease [10]. During the new triple procedure with toric IOL implantation, the corneal shape, especially of the posterior corneal curvature, can be expected to change [5, 11]. This may affect the accuracy of toric IOL power calculations.

The study presented here aims to assess the changes in magnitude and axis orientation of anterior, posterior, and total corneal astigmatism by means of the Pentacam Scheimpflug imaging system (Oculus, Wetzlar, Germany) in patients with FED after DMEK surgery.

## 2. Materials and Methods

This retrospective study included 29 eyes of 23 patients who underwent DMEK surgery at the Dept. of Ophthalmology, University of Muenster Medical Center. The study was prospectively approved by the local ethics committee and adhered to the tenets of the Declaration of Helsinki.

Patients were examined after attaining refractive stability (3–12 months after surgery) [5, 12] using rotating Scheimpflug corneal and anterior segment tomography (Pentacam HR; Oculus, Wetzlar, Germany). Only patients with complete pre- and postoperative Pentacam data with good quality corneal tomography scans were included. A skilled examiner performed Pentacam imaging; the automatic release mode of the Pentacam was used to minimize examiner-induced errors, and all patients were examined under the same conditions.

Eyes with a history of other corneal diseases, corneal infection or intraocular inflammation, trauma, corneal scars, contact lens wear four weeks before measurement, clinically significant graft detachment, or delayed corneal clearance were excluded. Some of the subjects had been included in our previous reports on refractive evaluation of patients with FED and patients after endothelial keratoplasty [11].

Corneal pachymetry at the apex and corneal topography data including average keratometry reading (Km^F^), average keratometry reading of the posterior corneal surface (Km^B^), and the magnitude and axis orientation of anterior, posterior, and total corneal astigmatism on the central 15° ring (equal to the 3.0 mm ring) were obtained and analyzed.

1. Anterior corneal astigmatism (AA) is the astigmatism value arising from the anterior corneal surface alone and calculated as the difference in simulated keratometry values (calculated using the standard keratometric index (1.3375) and the radius of anterior corneal curvature) between the steepest and the flattest meridians [13–16].
2. The posterior corneal astigmatism is defined as the difference in keratometry values between the flattest and steepest meridians, which were calculated using the radius of posterior corneal curvature and the refractive indices of the cornea (1.376) and aqueous humor (1.336) [13–16].
3. Total corneal astigmatism is defined as the difference in total corneal refractive power between the steepest and flattest meridians (calculated by ray tracing through the anterior and posterior corneal surfaces, according to Snell's law) [13–16].

For analysis of the relative orientations of corneal astigmatism, anterior corneal astigmatism, and total corneal astigmatism, these were classified as follows [13–15]:

1. with-the-rule: 60° < the steep meridian < 120°
2. against-the-rule: 0° < the steep meridian < 30° or 150° < the steep meridian < 180°
3. oblique: otherwise.

Since the dioptric power of the posterior corneal surface is negative, the posterior corneal astigmatism was classified as follows [13–15]:

1. with-the-rule: 0° < the steep meridian < 30° or 150° < the steep meridian < 180°
2. against-the-rule: 60° < the steep meridian < 120°
3. oblique: otherwise.

The vector difference between preoperative and postoperative posterior corneal astigmatism was calculated by vector analysis, as described by Holladay et al. [17].

All patients underwent standard DMEK surgery (between 2014 and 2015), performed by the same surgeon (LZ). The surgical procedure was described previously in details [1, 11].

### 2.1. Statistical Analysis

Data management was performed with Microsoft Excel 2010. IBM SPSS® Statistics 22 for Windows (IBM Corporation, Somers, NY, USA) was used for statistical analyses. The normality of the data distribution was tested using the Kolmogorov-Smirnov test. A normal distribution of the data was confirmed for Km^F^, anterior corneal astigmatism, total corneal astigmatism, Δ pachymetry at the apex, and the vector difference between preoperative and postoperative posterior corneal astigmatism. Parametric analysis (Student's *t*-test for paired values, Pearson's correlation coefficient (*r*_P_)) was then carried out. When the data did not fit a normal distribution (Km^B^, posterior corneal astigmatism), nonparametric tests (two-sided Wilcoxon signed-rank test, Spearman's correlation coefficient (*r*_S_)) were performed. Changes at follow-up compared with baseline were assessed, assuming left and right eyes of the same patient to be independent. Data are reported as mean ± standard deviation. The level of statistical significance was set at *P* ≤ 0.05. Inferential statistics are intended to be exploratory, not confirmatory, and were interpreted accordingly.

## 3. Results

The demographics of our population are summarized in Table 1. The corneal thickness at the apex and average keratometry reading of the posterior corneal surface (Km^B^) changed significantly after surgery (*P* < 0.001). The magnitudes of anterior, posterior, and total corneal astigmatism did not change significantly after DMEK (anterior astigmatism: before =1.14 ± 0.76 D, after =1.30 ± 0.77 D, *P* = 0.43; posterior astigmatism: before =0.38 ± 0.29 D, after =0.33 ± 0.20 D, *P* = 0.76; total astigmatism: before 1.64 ± 1.33 D; after =1.61 ± 0.90 D, *P* = 0.93).

**Figure 1:**
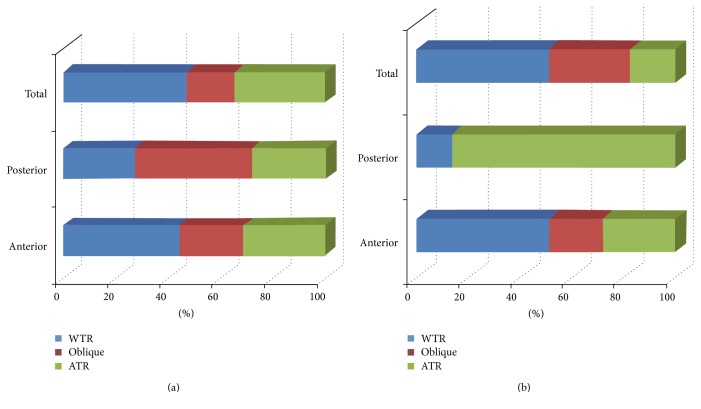
Distributions of anterior, posterior, and total corneal astigmatisms before (a) and after DMEK surgery (b). ATR: astigmatism against-the-rule; WTR: astigmatism with-the-rule; oblique: astigmatism oblique.

**Figure 2:**
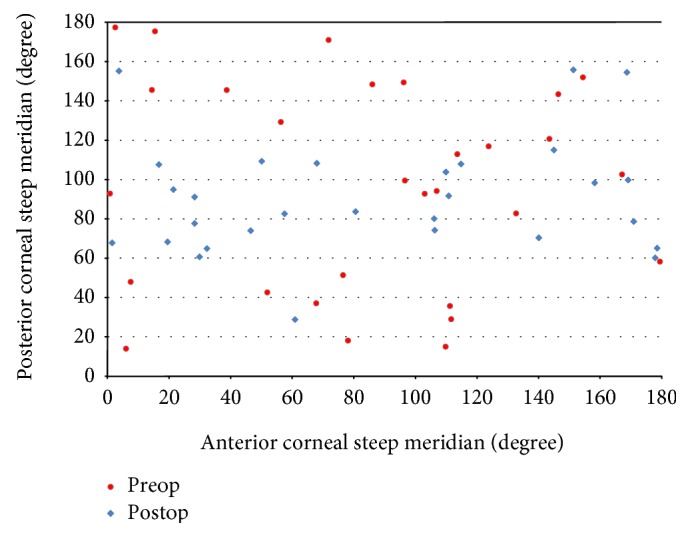
Location of steep meridian on anterior and posterior corneal surfaces before (red) and after surgery (blue) demonstrating the change in orientation of the posterior corneal astigmatism.

ATR astigmatism of the posterior corneal surface was found in 27.59% (*n* = 8) of patients before surgery and in 86.21% (*n* = 25) of patients after DMEK surgery. Figures 1(a) and 1(b) show the orientation of anterior, posterior, and total corneal astigmatism before (a) and after (b) DMEK surgery. The proportion of WTR astigmatism in anterior and total corneal astigmatism before and after surgery did not change markedly (Figure 1).  Figure 2 shows the location of the steep meridian on anterior and posterior corneal surfaces before and after DMEK surgery.

**Figure 3:**
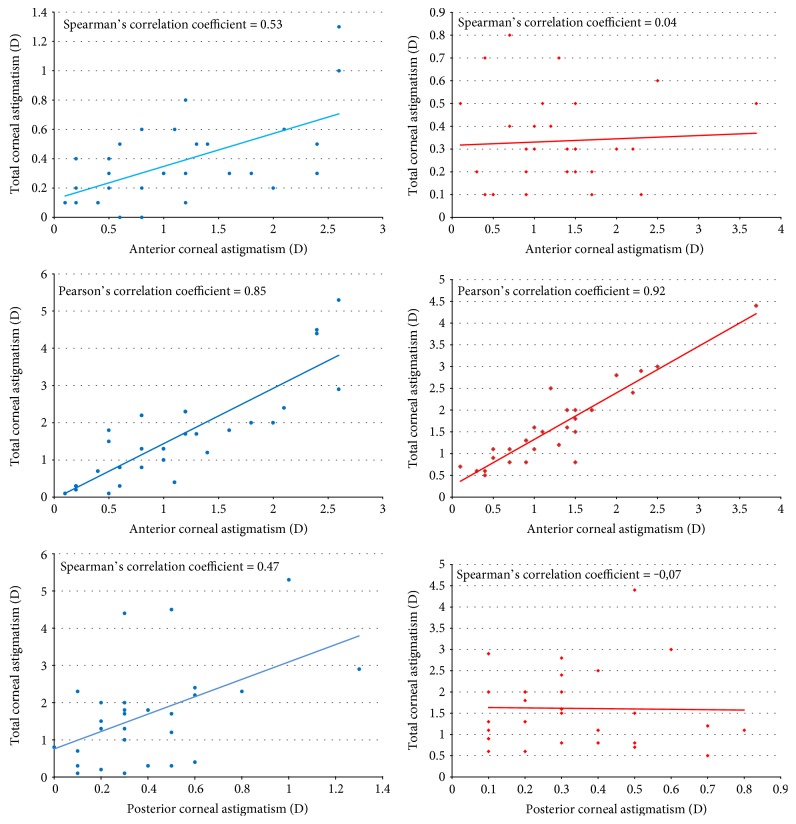
Showing correlations between the magnitudes of anterior posterior and total corneal astigmatism before (blue) and after (red) DMEK surgery. Before surgery, we found a significant correlation between the magnitudes of anterior and posterior corneal astigmatism (Spearman's correlation coefficient (*r*_S_) = 0.53, *P* = 0.003); after surgery, this correlation was not significant (*r*_S_ = 0.04, *P* = 0.843).

Before surgery, we found significant correlations between the magnitudes of anterior and posterior corneal astigmatism (*r*_S_ = 0.53, *P* = 0.003), between those of posterior and total corneal astigmatism (*r*_S_ = 0.47, *P* = 0.010), and between those of anterior and total corneal astigmatism (*r*_P_ = 0.85, *P* < 0.001). After surgery, the correlations between the magnitudes of anterior and posterior corneal astigmatism and between those of posterior and total corneal astigmatism were not significant, (*r*_S_ = 0.04, *P* = 0.843) and (*r*_S_ = −0.07, *P* = 0.721), whereas we found a strong correlation between the magnitudes of anterior and total corneal astigmatism (*r*_P_ = 0.92, *P* < 0.001) (Figure 3).

**Figure 4:**
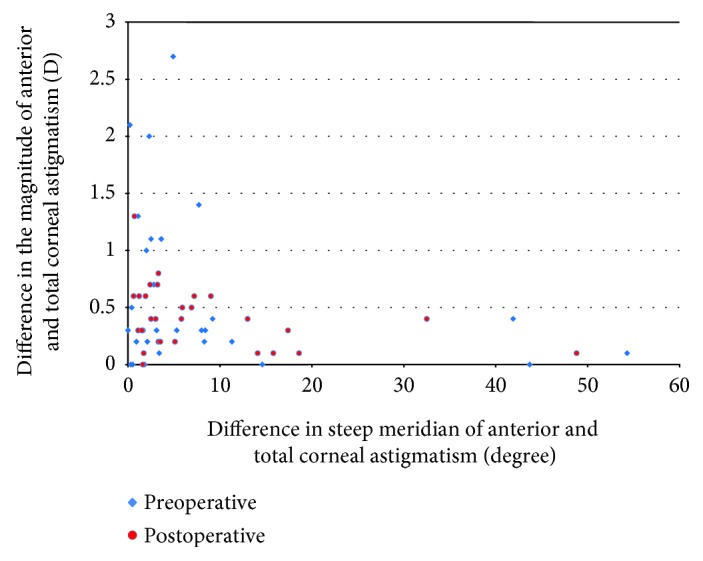
Showing the differences in magnitude and orientation between anterior and total corneal astigmatism before (blue) and after (red) DMEK. The figure illustrates the complexity of the estimation of total corneal astigmatism based on anterior corneal curvature. Note the large number of outliers.

Figure 4 shows the changes in the relationship of anterior to total corneal astigmatism before and after DMEK surgery and underlines the complexity of accurate estimation of posterior corneal astigmatism based on the measurement of the anterior corneal curvature in these cases.

**Figure 5:**
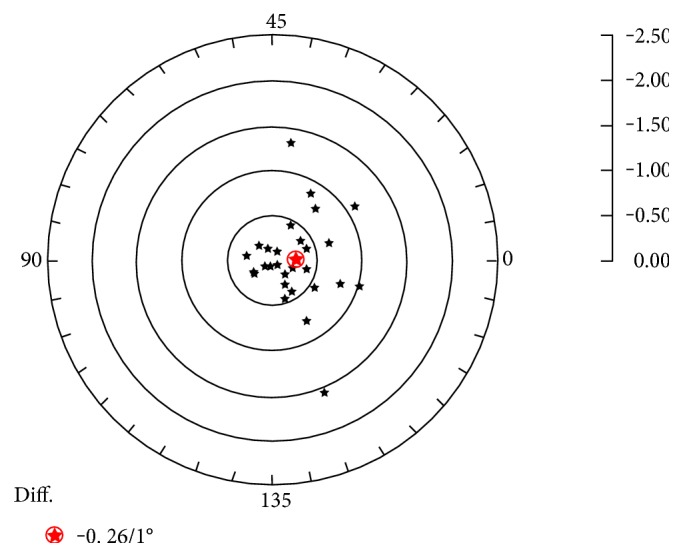
Doubled-angle plot for the vector difference between the posterior corneal astigmatism before and after DMEK. The red spot shows the centroid.

Figure 5 shows the doubled-angle plot for the vector difference between the posterior corneal astigmatism before and after DMEK.

Moreover, there was a significant correlation between the vector difference between preoperative and postoperative posterior corneal astigmatism and the change in corneal pachymetry at the apex (*r*_P_ = 0.47, *P* = 0.010).

## 4. Discussion

Toric IOLs have been developed to reduce corneal astigmatism at the time of cataract surgery. Owing to the excellent visual outcome and refractive stability achieved after DMEK [5, 12], (toric) IOL power calculations in patients after phakic DMEK or within the setting of the new triple procedure are becoming a topic of much interest in clinical practice [18, 19]. As previously described, the change in corneal refractive power after DMEK is based mainly on changes in the posterior corneal curvature, whereas the anterior corneal curvature does not alter significantly [5, 11]. The importance of posterior corneal astigmatism has recently been highlighted in various studies [20–24]. Toric IOL power calculations based on the anterior corneal curvature, ignoring posterior corneal astigmatism, can over- or underestimate total corneal astigmatism [20–24].

Different studies have shown that with-the-rule is the most common type of anterior corneal astigmatism among younger individuals, whereas against-the-rule astigmatism becomes more common with aging [14, 25]. Posterior corneal astigmatism remains ATR with aging [14, 25]. In this study, our results demonstrate that the mean magnitudes of anterior and total corneal astigmatism did not change significantly after DMEK surgery. There was no significant change in the magnitude of posterior corneal astigmatism either. Our results are in line with previous findings concerning the magnitude of posterior corneal astigmatism in normal eyes and in eyes with FED [10, 13, 16, 25, 26].

We know that in eyes with WTR astigmatism of the anterior corneal surface, the presence of ATR astigmatism of the posterior corneal surface compensates for anterior corneal astigmatism and thus reduces total corneal astigmatism. However, in eyes with ATR astigmatism of the anterior corneal surface, it increases total corneal astigmatism [15, 26]. Wacker et al. showed that eyes with advanced FED were more likely than normal eyes to have oblique or WTR posterior corneal astigmatism [10]. In this study, we found that the posterior corneal surface shifts back from with-the-rule to against-the-rule astigmatism after surgery in most cases, whereas no marked change was observed in the orientation of the anterior or total corneal astigmatism. The vector difference between preoperative and postoperative posterior corneal astigmatism correlates with the change in corneal thickness. These findings will be helpful for calculation of toric IOL power within the setting of the triple procedure.

Yokogawa et al. demonstrated in small case series (10 patients) that triple DMEK procedure with toric IOL implantation provided relatively good UDVA and reduced refractive astigmatism. However, compared with simple cataract surgery with toric IOL implantation, the postoperative corneal astigmatism may not be as predictable as in patients undergoing cataract surgery alone [19]. This study and our results demonstrate that the calculation of toric IOL power in the setting of new triple procedure is complicated by the expected changes in corneal curvature. In cases with minimal or mild preoperative corneal swelling, it is advisable, given the impact of posterior corneal astigmatism [21–24], to acquire extensive biometric data from the entire cornea and consider the orientation and magnitude of a measured posterior corneal astigmatism and to factor in the changes demonstrated here. For assessment for the new triple procedure in cases with severe preoperative corneal swelling, it would be difficult to predict changes in corneal astigmatism. The large number of outliers, as shown in Figure 4, should be taken into account when deciding whether to plan a toric IOL implantation or rather, in critical cases, to go with a standard IOL. The other possible way is to perform phakic DMEK and then to perform cataract surgery after corneal stabilization; in this case, the risk of endothelial cell loss caused by secondary cataract surgery has to be taken into account. For more accurate prediction of corneal astigmatism after DMEK, the deswelling profile of the cornea and factors that might influence these changes needs to be evaluated in detail.

For calculation of toric IOL power after phakic DMEK, given the difference in magnitude and axis orientation between anterior and total corneal astigmatism seen in our population (Figure 4), we recommend a calculation based on a measured anterior and posterior corneal curvature after attainment of refractive stability, compared with population-averaged value. In our opinion, this would improve the accuracy of toric IOL power calculations in these particular patients.

In patients with FED, we found a significant correlation between the magnitude of the anterior and posterior as well as posterior and total corneal astigmatism at the steepest corneal meridians. This correlation was comparable to findings previously described for normal or keratoconic corneas [14–16], suggesting that the role of the posterior cornea may be more critical in cases with a high anterior or total corneal astigmatism. After DMEK, the correlations between posterior/anterior and between posterior/total corneal astigmatism were not significant. Furthermore, the correlation coefficient between anterior and total corneal astigmatism after surgery was higher than before.

This study is burdened with some limitations, which may have affected the results to some extent. First, the follow-up time after surgery (3–12 months) was not exactly defined. However, refractive stability for 3 months after DMEK has been described previously in the literature [5, 12]. Second, it was performed in a retrospective design. Third, we included a relatively small number of eyes. However, further studies in a prospective setting with detailed long-term analysis, especially of the posterior corneal curvature before and after DMEK, are urgently needed.

In conclusion, posterior corneal astigmatism should be considered for more accurate total corneal astigmatism predictions. It should be noted that the orientation of the posterior corneal astigmatism and the relationship of anterior to total corneal astigmatism appear to change after DMEK surgery, whereas the magnitude of the posterior corneal astigmatism does not change significantly. The vector difference between preoperative and postoperative posterior corneal astigmatism correlates with the change in corneal thickness.

Finally, the essential point to emphasize is that the power of astigmatism may change as a result of DMEK surgery. However, attention should also be paid to possible changes in axis orientation. These simple findings are clinically very important for the calculation of toric IOL power within the setting of the new triple procedure and in patients after phakic DMEK.

## Figures and Tables

**Table 1:** Demographics of the study population in eyes after Descemet membrane endothelial keratoplasty. Mean ± standard deviation (minimum–maximum). Bold: statistically significant results.

	Before surgery	After surgery	*P* value
Subjects	29 eyes (23 patients)		
Age (years)	67.55 ± 9.83 (47–83)		
Gender (male: female)	10 : 13		
Pachymetry at the apex (*μ*m)	621.21 ± 68.21 (486–895)	510.41 ± 34.04 (443–593)	**<0.001**
Km^F^ (D)	43.33 ± 1.43 (40.9–46)	43.11 ± 1.41 (41.2–46.3)	0.09
Km^B^ (D)	−5.73 ± 0.67 (−3.1–−6.7)	−6.32 ± 0.28 (−5.9–−7)	**<0.001**
Anterior corneal astigmatism (D)	1.14 ± 0.76 (0.1–2.6)	1.30 ± 0.77 (0.1–3.7)	0.43
Posterior corneal astigmatism (D)	0.38 ± 0.29 (0.0–1.3)	0.33 ± 0.20 (0.1–0.8)	0.76
Total corneal astigmatism (D)	1.64 ± 1.33 (0.1–5.3)	1.61 ± 0.90 (0.5–4.4)	0.93
